# Species richness, extent and potential threats to mangroves of Sarangani Bay Protected Seascape, Philippines

**DOI:** 10.3897/BDJ.11.e100050

**Published:** 2023-03-28

**Authors:** Angelo Rellama Agduma, Kun-Fang Cao

**Affiliations:** 1 State Key Laboratory for Conservation and Utilization of Subtropical Agrobioresources, Guangxi University, Nanning 530004, Guangxi, China State Key Laboratory for Conservation and Utilization of Subtropical Agrobioresources, Guangxi University Nanning 530004, Guangxi China; 2 Guangxi Key Laboratory of Forest Ecology and Conservation, College of Forestry, Guangxi University, Nanning 530004, Guangxi, China Guangxi Key Laboratory of Forest Ecology and Conservation, College of Forestry, Guangxi University Nanning 530004, Guangxi China; 3 Ecology and Conservation Research Laboratory, Department of Biological Sciences, College of Science and Mathematics, University of Southern Mindanao, Kabacan 9407, Cotabato, Philippines Ecology and Conservation Research Laboratory, Department of Biological Sciences, College of Science and Mathematics, University of Southern Mindanao Kabacan 9407, Cotabato Philippines; 4 Environmental Conservation and Protection Center, Provincial Capitol Compound, Alabel 9501, Sarangani, Philippines Environmental Conservation and Protection Center, Provincial Capitol Compound Alabel 9501, Sarangani Philippines

**Keywords:** Coastal biodiversity, Mindanao, occurrence, remote sensing, Sarangani, true mangrove

## Abstract

Mangroves form one of the most vital tropical ecosystems that support many species and surrounding communities. The Sarangani Bay Protected Seascape (SBPS) in the south of Mindanao Islands in the Philippines is home to a large number of mangrove species, which have not been fully explored. We updated the list of true mangrove species for SBPS from 10 to 24 by integrating the results of our survey and other past mangrove assessments. A practical spatial analysis approach was used to estimate the current mangrove forest extent of SBPS at 514 ha, as compared to 479 ha and 332 ha in 1998 and 2016, respectively, from other independent reports. Mangrove cover was negatively related to built area, cropland, bare ground, rangeland and total human population, but positively related to the number of fishing boats and total tree cover. In addition, we identified other potential anthropogenic threats to mangroves and categorised them into forest clearing or deforestation, over-extraction and pollution. The benefits of mangrove cover expansion, adoption of mangrove-friendly aquaculture and revitalising degraded mangrove forests outweigh their constraints. Our work provided a locally relevant understanding of the potential causes of mangrove loss and the values of human actions in mangrove dynamics, which will contribute to reliable and informed decision-making for the conservation of mangrove species and restoration of mangrove forests in SBPS.

## Introduction

Mangroves thrive in saline and anaerobic tidal flats and banks of rivers and seas in tropical and subtropical coastal zones of the world (Friess et al. 2019). They were once regarded as useless wastelands (Spalding et al. 1997), but are now being recognised to perform critical ecosystem processes and provide many ecosystem services. They support the cycle of nutrients and carbon, help maintain adjacent ecosystems and protect coastal areas, together with other direct services for the survival and welfare of coastal communities (Spalding et al. 1997, Hamilton and Friess 2018, Alongi 2020). Despite these benefits, mangroves continually face a rapid decrease in cover extent and decline in habitat quality driven by anthropogenic and stochastic threats throughout their range (Valiela et al. 2001, Gilman et al. 2008, Feller et al. 2010, Polidoro et al. 2010, Donato et al. 2011, Primavera et al. 2016b, Friess et al. 2019, Goldberg et al. 2020). The original mangrove forests of the world had been reduced by approximately 35% in the twentieth century and were subjected to a worldwide mean deforestation rate of approximately 2.07% per year (Valiela et al. 2001). About 3.4% loss per year was documented from 1996 to 2020, which was approximately twice that of worldwide gain in mangrove areas (Bunting et al. 2022). Six of the 10 nations with the highest total areal mangrove loss are in Asia, including the Philippines (Bryan-Brown et al. 2020). In 1920 the Philippines had 450,000 ha of mangrove forests but lost about 317,500 ha by 1990 (Primavera 1995). Recent estimates of Philippine mangrove cover were approximately 256,185 ha in the year 2000 (Long and Giri 2011) and 240,824 ha in 2010 (Long et al. 2014) using Landsat satellite data, while in 2019, the national mangrove area was estimated at 227,808 ha (Neri et al. 2021) using Sentinel 2-based mangrove vegetation index (MVI) (Baloloy et al. 2020). However, the Forest Management Bureau of the Department of Environment and Natural Resources (DENR-FMB) of the Philippines estimated the country’s mangrove cover at 311,400 ha in 2020 (FMB 2021).

The most comprehensive inventory of Philippine mangrove species is probably that of Primavera et al. (2016a). They identified 33 true mangroves, that is, exclusive to the intertidal saline zones (Quadros et al. 2021), including *Rhizophora* x *lamarckii*, a hybrid of *R.apiculata* and *R.stylosa*. This differs from the earlier report of Fernando and Pancho (1980) which listed 39 species and one variety, while Calumpong and Meñez (1997) included 39 species in their account. To date, substantial development in the inventory of Philippine mangroves has been made. Some species were removed, while others were renamed or added to the list. For example, *Acrostichum* spp., *Heritieralittoralis* and *Excoecariaagallocha* were classified as mangrove associates, i.e. non-exclusive to mangrove forest (Quadros et al. 2021), based on ecological, osmotic and leaf properties (Wang et al. 2011). Primavera et al. (2004) previously regarded *Aegialitisannulata* and *H.littoralis* as true mangroves, but these are now reclassified, based on the criteria set by Wang et al. (2011), although *E.agallocha* has been retained as a true mangrove in the Philippines (Primavera et al. 2016a). Calumpong and Meñez (1997) did not include *Camptostemonphilippinense* in their mangrove species list for the Philippines, while Fernando and Pancho (1980), Primavera et al. (2004) and Primavera et al. (2016a) included the species in their records. Morphological and molecular evidence shows that *Ceriopsdecandra* and *C.zippeliana* are distinct species and the latter is the one that is found in the Philippines (Sheue et al. 2009). Therefore, Primavera et al. (2016a) corrected the widely known *C.decandra* in Aklan Panay Province as *C.zippeliana*.

The Sarangani Bay Protected Seascape (SBPS), located in the southernmost part of Mindanao Islands in the Philippines, is home to a large number of mangrove species, yet it is not well-explored. The mangroves of SBPS mostly form narrow fringes and patches parallel to the shoreline in rocky, sandy or riverine areas. Some grow in between taller trees such as coconut and other beach forest species and are interspersed with small houses of coastal dwellers along the shore and mudflats. No detailed taxonomic and ecological accounts, as well as their distribution, are publicly available for the mangroves in the area. For example, information on mangrove species diversity in SBPS is limited to specific mangrove stands and localities only (Mullet et al. 2014, Natividad et al. 2014, Natividad et al. 2015, Barcelete et al. 2016, Bigsang et al. 2016, Lagnason et al. 2016, Jumawan 2022). Indeed, the total number of mangrove species in SBPS is unknown, scattered and unconsolidated. Furthermore, SBPS is not included in the global map of mangrove extent of Global Mangrove Watch version 3.0 (Bunting et al. 2022). The SBPS was also missed out on the 2019 Philippine Mangrove Extent Map using the MVI (Baloloy et al. 2020) due to several limitations (Neri et al. 2021). Moreover, mangroves in SBPS are not spared from various potential threats which are sparsely documented.

This study aimed to database true mangrove species in SBPS; map the extent of mangrove forests; and examine the potential threats to mangroves in SBPS. This is to construct a mangrove diversity profile of SBPS, which will aid in better understanding what frame the structure, processes and services of the mangrove forests. It will facilitate further comprehensive studies to reduce the gap in our current understanding of the mangrove flora in the area and will provide information on the mangrove ecosystem health towards a well-informed conservation priority and management in SBPS.

## Materials and Methods

### Study area

**Figure 1.**
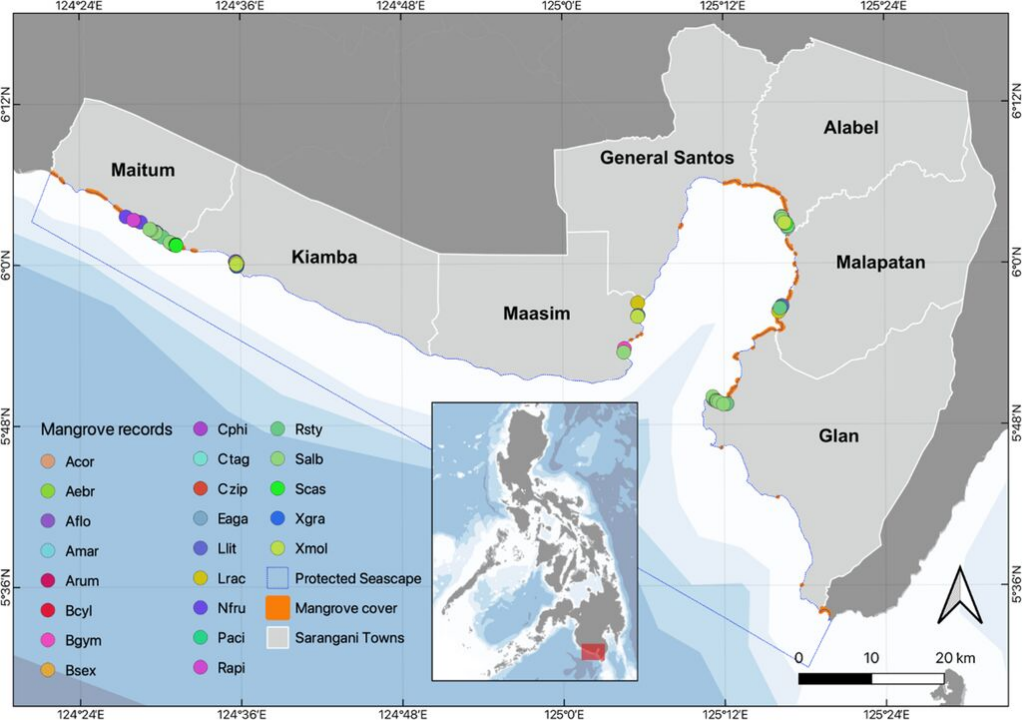
Occurrence of mangrove species and mangrove cover extent along the coast of Sarangani Bay Protected Seascape, Philippines. Acor (*Aegicerascorniculatum*), Aebr (*Acanthusebracteatus*), Aflo (*Aegicerasfloridum*), Amar (*Avicenniamarina*), Arum (*Avicenniarumphiana*), Bcyl (*Bruguieracylindrica*), Bgym (*Bruguieragymnorrhiza*), Bsex (*Bruguierasexangula*), Cphi (*Camptostemonphilippinense*), Ctag (*Ceriopstagal*), Eaga (*Excoecariaagallocha*), Llit (*Lumnitzeralittoralis*), Lrac (*Lumnitzeraracemosa*), Nfru (*Nypafruticans*), Paci (*Pemphisacidula*), Rapi (*Rhizophoraapiculata*), Rsty (*Rhizophorastylosa*), Salb (*Sonneratiaalba*), Scas (*Sonneratiacaseolaris*), Xgra (*Xylocarpusgranatum*), Xmol (*Xylocarpusmoluccensis*) and Xrum (*Xylocarpusrumphii*). The georeferenced mangrove distributions are provided in Suppl. material 1, which can also be accessed through the Global Biodiversity Information Facility (GBIF) network (Agduma and Cao 2023).

The SBPS is located between 5°33’25” and 6°6’15” N and 124°22’45” and 125°19’45” E in the south of Mindanao, Philippines, bordered by the Sarangani Province and General Santos City, hereafter "SarGen" (Fig. 1). The SBPS has a total area of 215, 950 ha and a coastline of 218.18 km. The climate is monsoonal, with the influences of the northeast monsoon from November to March and the southwest monsoon from June to October. The mean annual precipitation is 960 mm and is evenly distributed throughout the year. The mean annual temperature of the area is 27.85°C, with a mean annual relative air humidity of 79.38% (Emperua et al. 2018, USAID Oceans 2019). The SBPS sea water has a mean pH of 8.16 and a mean salinity of 23.80 parts per thousand (ppt). Its mean annual nitrate content is 0.21 mgl^-1^, while its phosphate content is 0.15 mgl^-1^ (data from Department of Environment and Natural Resources-Environment Management Bureau, Region 12, Philippines).

### Survey and identification of mangroves

A prior informed consent from the National Commission of Indigenous Peoples, a permit to study through the Sarangani Bay Protected Seascape Protected Area Management Board resolution no. 2017-053, s. 2017 and certification control no. SBPS-2017-046 and a gratuitous permit (no. 284) through the Biodiversity Management Board of the Department of Environment and Natural Resources, Republic of the Philippines were secured. Only true or exclusive mangroves following the classification of Primavera et al. (2016a), based on the criteria of Wang et al. (2011), were the subjects of this study. Primavera et al. (2016a) identified 33 true mangroves in the Philippines. From this general list, we created the true mangrove list for SBPS by a complete inventory of mangrove species at known mangrove sites along the coast of SBPS from January 2018 to December 2019 and June to October 2022. Additionally, mangrove diversity data from previous surveys (Mullet et al. 2014, Natividad et al. 2014, Natividad et al. 2015, Barcelete et al. 2016, Bigsang et al. 2016, Lagnason et al. 2016, Jumawan 2022) were also used for the list of mangrove species for SBPS. The conservation status of the mangroves was determined using the International Union for the Conservation of Nature Red List (IUCN 2022-1) (IUCN 2022). Furthermore, the national level conservation status of the species was determined according to the Philippines’ National List of Threatened Flora as specified in the Department of Environment and Natural Resources Administrative Order No. 2017-11 (DENR 2017).

### Mapping mangrove extent, land-use cover and potential threats

We followed a similarly practical approach to mapping mangroves as that of Altamirano et al. (2010) with modifications to map the extent of mangrove cover on the coastlines of SBPS. The boundaries of known mangrove sites were initially tracked using a global positioning system (GPS, Etrex 201x, Garmin Ltd., Kansas, USA) during the mangrove species surveys. Using the geographical information, the mangrove areas were drawn and digitised in the Google Earth Pro environment in order to construct the mangrove extent polygons. To determine the extent of mangrove areas and map mangrove sites detected, but not visited previously, we compared the characteristics of Google Earth images with aerial images available from previous studies (e.g. Natividad et al. 2014, Natividad et al. 2015, Barcelete et al. 2016, Bigsang et al. 2016, Lagnason et al. 2016, Baloloy et al. 2020, Faustino et al. 2020, Neri et al. 2021, Jumawan 2022). From June to October 2022, we conducted a ground-truth sampling to validate the mangrove layers created and the suspected mangrove sites based on aerial images. Then, all the mangrove layers were cleaned and curated. The KML (key-hole mark-up language) versions of the mangrove layer were imported to QGIS (version 3.26) to measure the extent of the mangrove forests (ha) and the length (km) of the mangrove extent. The areas and lengths of the mangrove forests were then measured according to the political boundaries of the coastal areas in SBPS. A confusion matrix is provided to substantiate the accuracy of the spatial analysis (overall accuracy = 0.94, Kappa coefficient = 0.88) (Suppl. material 5). Ten-m resolution land-use/land-cover (LU/LC) data generated from Karra et al. (2021) was used to determine land-use cover. Using QGIS (WGS 84), the land cover classes, such as trees, built areas, crops, bare ground, flooded vegetation, water and rangeland, within each political boundary were determined. ‘Trees’, hereafter will be called total tree cover, which refers to “any significant clustering of tall (~ 15 feet or higher) dense vegetation, typically with a closed or dense canopy; examples: wooded vegetation, clusters of dense tall vegetation within savannahs, plantations, swamp or mangroves (dense/tall vegetation with ephemeral water or canopy too thick to detect water underneath)” (Karra et al. 2021). We used the land area occupied by built areas, cropland, bare ground and rangeland, derived above, together with the total human population for the year 2020 (PSA 2021) and the number of boats (Emperua et al. 2018) in every town or city as proxies of potential threats. Then, the relationships of mangrove cover to total tree cover and proxies to potential threats were determined using Spearman’s rho (ρ) correlation in R, version 4.2.2 (R Core Team 2022). Other perceived potential threats to mangroves were noted during site surveys.

## Results

### Status and distribution of mangroves in SBPS

**Figure 2.**
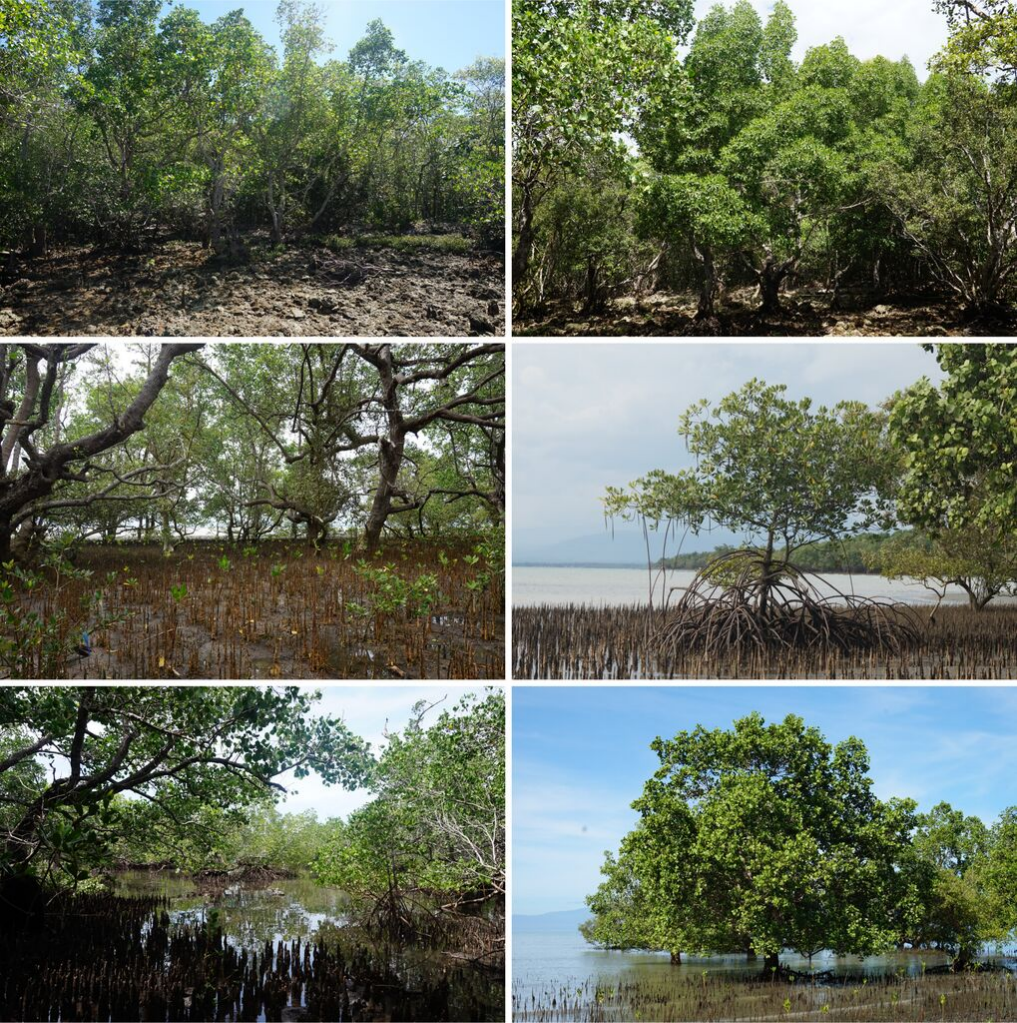
Mangrove forest types and some mangrove species in Sarangani Bay Protected Seascape, Philippines, Left (mangrove forest types): Top - Rocky; Middle - Sandy; Bottom - Basin, Right (mangroves): Top - *Bruguieracylindrica*; Middle - *Rhizophorastylosa*; Bottom - *Sonneratiaalba*.

There were 24 true mangroves recorded within SBPS from 10 families and 13 genera (Table 1). This is approximately 73% of the total true mangroves, 33 species, recorded for the Philippines (Primavera et al. 2016a). Twenty-two of these were documented in our survey, while other previous works identified 19 species. We noted additional distribution records of five species in SBPS in our study, namely *Aegicerascorniculatum*, *Camptostemonphilippinense*, *Lumnitzeralittorea*, *Rhizophorastylosa* and *Sonneratiacaseolaris*. Three species are listed as threatened on the International Union for Conservation of Nature Red List (IUCN 2022-1) (IUCN 2022). *Camptostemonphilippinense* is currently on the Endangered (EN) list, while *Avicennialanata* and *Avicenniarumphiana* are listed as Vulnerable (VU). *Aegicerasfloridum* is listed as near threatened (NT), while other remaining species are classified by IUCN as least concern (LC). The Philippines’ National List of Threatened Flora, specified in the Department of Environment and Natural Resources Administrative Order No. 2017-11, identified *C.philippinense* and *Pemphisacidula* as the only locally threatened mangroves and are placed under the EN category, while all other species are classified as Other Wildlife Species (OWS) (DENR 2017). The OWS is defined as “non-threatened species, subspecies, varieties or other infraspecific categories that have the tendency to become threatened due to destruction of habitat or other similar causes” (DENR 2017). The occurrence and distribution of mangroves are shown in Fig. 1 and Suppl. material 1. They can also be accessed through the Global Biodiversity Information Facility (GBIF) network (Agduma and Cao 2023). Representative photographs of mangroves in the study site are also shown in Fig. 2.

### Mangrove map and cover extent

Fig. 1 shows the mangrove extent map for SBPS, while the measured mangrove cover extent and length of the mangrove extent of the coastal towns are reflected in Table 2. Maitum and Glan have the longest extent of mangrove forests with 12.67 km and 11.07 km, respectively. However, Maitum has 60.01% of its coast covered by mangroves, while only 19% of the shoreline in Glan is covered by mangroves. Almost 68% of the coast of Alabel is lined by mangrove forests, the highest in the entire SarGen. Of the 40-km coastline of Kiamba, only 6% of it is occupied by mangroves. In terms of mangrove extent, Maitum has the largest area, with 138 ha contributing to 26.89% of the total mangrove area estimated for SBPS, followed by Glan with 129 ha, while General Santos City and Maasim have the least mangrove extent with 37 ha and 29 ha, respectively. In addition, it was revealed that Alabel has the largest mangrove area relative to the length of its coast (7.63 ha/km).

### Potential threats to mangroves in SBPS

**Figure 3.**
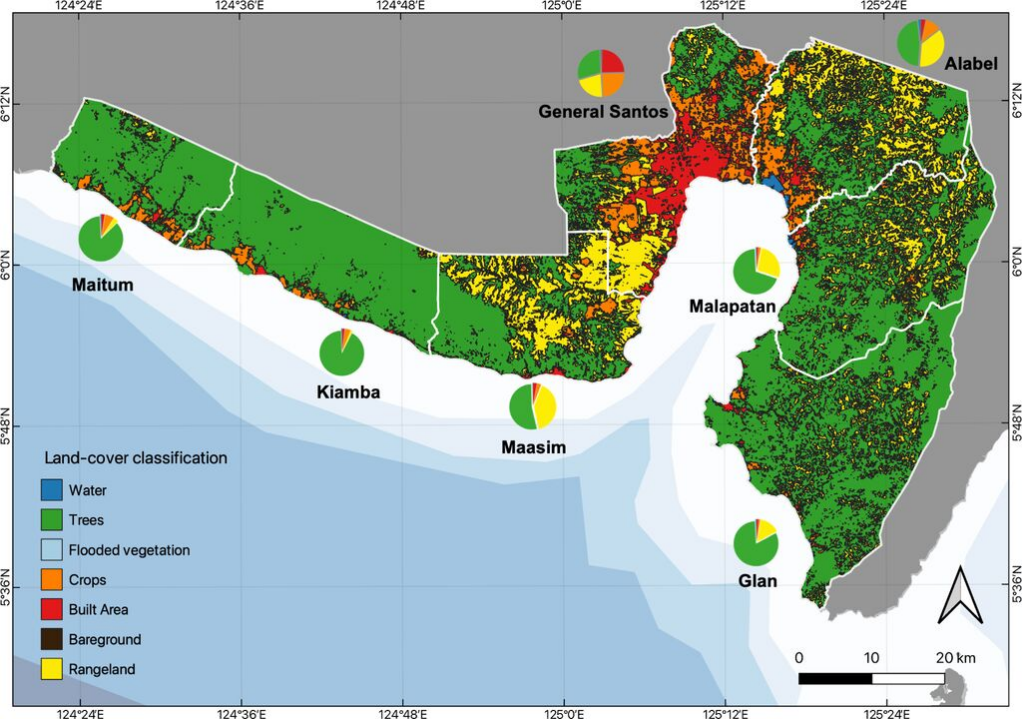
Land-use/land-cover proportions in every town/city around Sarangani Bay Protected Seascape, Philippines.

**Figure 4.**
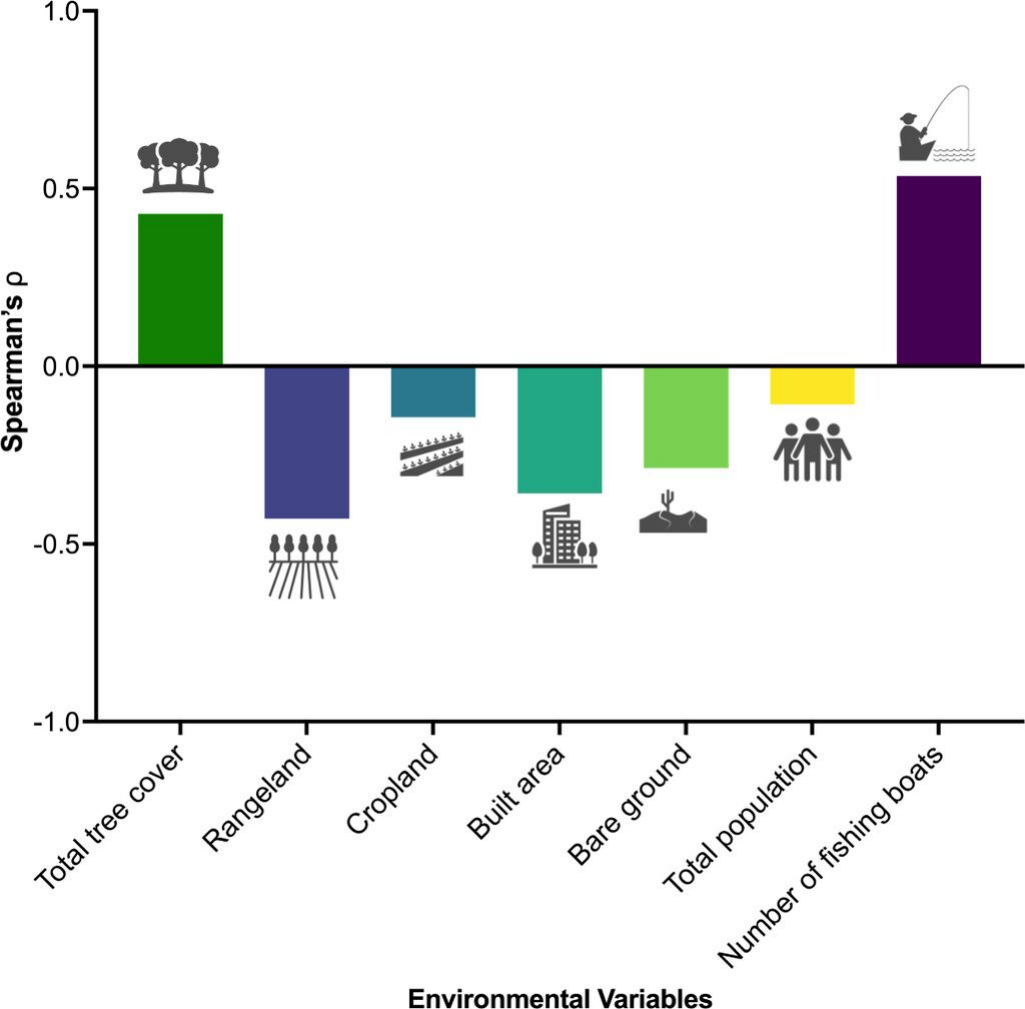
Relationship (Spearman’s ρ) of mangrove cover with total tree cover and some potential threats to mangroves in Sarangani Bay Protected Seascape, Philippines.

We believe that land-use change plays an important role in mangrove diversity and distribution. Here, we determined which of the different land-use classifications, based on the European Space Agency (ESA) Sentinel-2 (Karra et al. 2021), occupy the largest areas within SarGen (Fig. 3, Suppl. material 2). The largest area is occupied by total tree cover followed by rangeland. However, cropland and built area are also markedly high, especially in General Santos City and Alabel. It was revealed that cropland, built area, bare ground, rangeland and the total human population had negative relationships with mangrove cover, while the relationships of mangrove cover with the number of fishing boats and total tree cover were positive. However, all correlations were not statistically significant (Fig. 4, Suppl. material 3).

Moreover, the observed potential anthropogenic threats to mangroves in SBPS were classified into: (1) forest clearing, (2) over-extraction and (3) pollution. Clearing of mangrove forests in SBPS makes way for the construction of commercial establishments, canneries, residential settlements, aquaculture ponds (shrimp and fish), agriculture production (rice, corn and coconut), tourism and recreation and infrastructure (roads, bridges, ports, fishing wharves etc.). Additionally, the inhabitants of the area extract mangroves for fuelwood, charcoal and timber and as ornamental plants ('*bonsai*'). Potential pollution of seawater threatens mangroves as well from oil, solid wastes, silt, pesticides, fertilisers, effluents from aquaculture, livestock, domestic and urban areas and smoke from charcoal production.

## Discussion

### Mangrove species richness

The primary aim of this work was to generate a list of true mangrove species for SBPS by integrating the results of our survey and previous reports. Ten species were reported by de Jesus et al. (2001) and Alcala et al. (2008) along Sarangani Bay (Glan, Malapatan, Alabel, General Santos City and a portion of Maasim), but only eight of these were exclusive to the mangrove ecosystem (Primavera et al. 2016a). Subsequent works focused only on specific mangrove stands and localities along the coast of SBPS (Sarangani Bay plus the remaining parts of Maasim, Kiamba and Maitum). We summarised the species and their distribution in the coastal areas that line SBPS, based on publicly available assessments (Mullet et al. 2014, Natividad et al. 2014, Natividad et al. 2015, Barcelete et al. 2016, Bigsang et al. 2016, Lagnason et al. 2016, Jumawan 2022) and our survey (Table 1). Mullet et al. (2014) documented 13 true mangroves in Malapatan and reported for the first time *A.rumphiana*, *Bruguieracylindrica*, *B.gymnorrhiza*, *Xylocarpusgranatum*, *X.mollucensis* and *Nypafruticans* in SBPS, which are important additions to the list. Natividad et al. (2014) and Natividad et al. (2015) evaluated selected sites in Maasim and Alabel and reported 12 species that added *Ceriopstagal* and *L.racemosa* to the SBPS mangrove list. Lagnason et al. (2016) noted six mangroves, including *A.lanata*, in Kawas Marine Sanctuary in Alabel. However, this species had never been previously reported in the area and the Philippines lies outside its distribution range as previously reported (Chua 1998). Approximately the same year, Barcelete et al. (2016) and Bigsang et al. (2016) studied mangroves at other sites and the former documented another new species record for SBPS, *B.sexangula*, in Glan. Furthermore, Jumawan (2022) reported the same species as that of Natividad et al. (2014) and Natividad et al. (2015), but with one addition, *X.mollucensis*, in Alabel, whereas five true mangrove species were newly reported by the present survey in SBPS. Therefore, the cumulative true mangrove species tally for SBPS increased to 24 species from previous studies and our data. The highest true mangrove species richness was documented in Malapatan and Alabel, while General Santos City had the lowest mangrove record of species.

Previous studies of Jumawan (2022) and Mullet et al. (2014) reported *C.decandra* in SBPS, particularly in Alabel and Malapatan, while we found samples of the species in Alabel only. Additionally, Natividad et al. (2014)and Natividad et al. (2015) found the species, along with *B.cylindrica* and *P.acidula*, outside of their sampling plots. However, it is not clear at which study site, Alabel or Maasim, they were found; hence, we added the three species to the Alabel as well as to the Maasim list. *Ceriopszippeliana* is found in the Malay Peninsula, Singapore, Bintan Island, Thailand, Vietnam, Borneo, Java, Sulawesi, Lesser Sunda Islands, Moluccas and the Philippines, while *C.decandra* occurs in India, Bangladesh, Myanmar and Thailand (Sheue et al. 2009). Consequently, Primavera et al. (2016a) updated the name of *C.decandra* to *C.zippeliana* in their book, Mangroves and Beach Forest Species in the Philippines. This misidentification is not surprising because the two species closely resemble each other, based on recent morphological and phylogenetic analyses (Ruang-areerate et al. 2022). Therefore, this study also updates the name of *C.decandra* in SBPS to *C.zippeliana*, until the emergence of further evidence that will prove otherwise. The new species distribution records for SBPS were found in Kiamba, Maitum, Maasim and Malapatan. *Aegicerascorniculatum* thrives abundantly in a riverine/estuarine mangrove forest in Nalus, Kiamba, while a mangrove site in Kiambing, Maitum is a sanctuary for *S.caseolaris*. On the other hand, a small population of *L.littorea* grows in Tinoto, Maasim, as well as in Pananggalon, Poblacion, Malapatan together with the endangered *C.philippinense*. Remarkably, none of the previous surveys recorded *R.stylosa*. We found that this species is one of the most widespread taxa in SBPS along with *R.apiculata* and *Sonneratiaalba*. Furthermore, most of the previous studies identified *R.mucronata* at their study sites. These recent findings support the call for more comprehensive surveys on mangrove diversity in SBPS clarifying the identity and distribution of *A.lanata*, *C.zippeliana*, *R.stylosa* and *R.mucronata*. The possibility that new species, new distribution records and other amendments to our species list (Table 1) are expected in future studies.

### Mangrove areal extent

**Figure 5.**
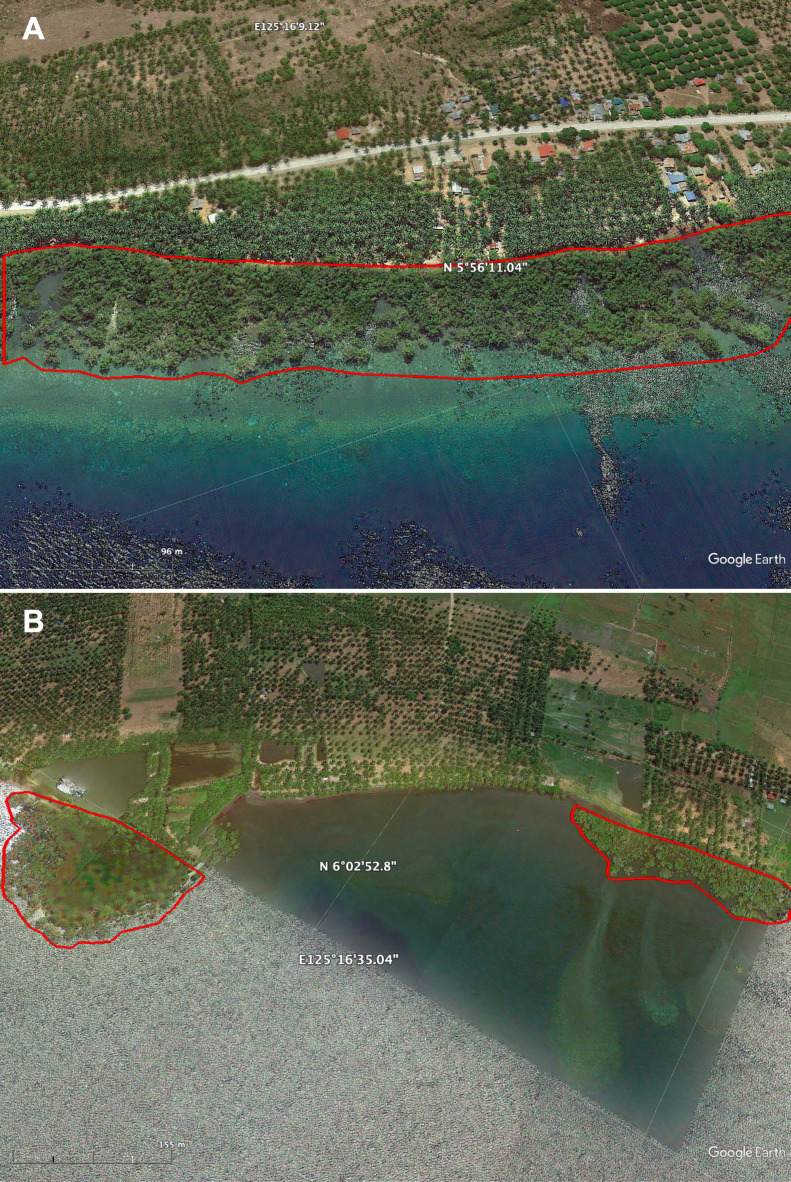
Examples of (A) fringing and (B) patchy mangrove forests in Sarangani Bay Protected Seascape, Philippines.

Bunting et al. (2022) found that the extent of mangroves in the Philippines decreased by 7,934 ha between 1996 and 2020. However, in this global map of mangrove extent, the mangroves in SBPS were not included. The MVI developed by Baloloy et al. (2020), which was used to generate the 2019 Philippine Mangrove Extent Map, also missed the mangroves in SBPS (Neri et al. 2021). Some structural and environmental constraints affect the detectability of mangroves with remote sensing models. For example, the sparse canopy and short stature of mangroves relative to other trees cause their limited visibility (Hickey and Radford 2022). The mangroves in SBPS form narrow fringes and small patches of stands (Fig. 5), while some grow in between houses of dwellers and taller trees along the coast. Tidal inundation can also affect the spectral signatures of the mangroves (Neri et al. 2021) such that the spectra of the mangroves and the water during high tide are the same (Hu et al. 2020).

**Figure 6.**
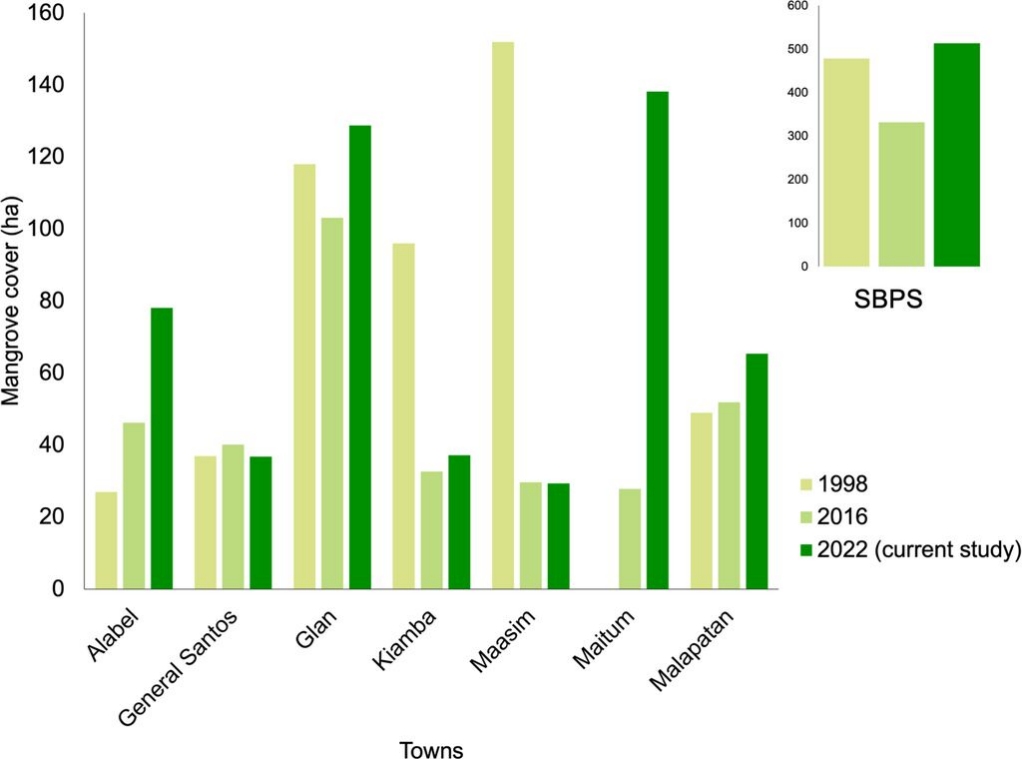
Total mangrove cover of every coastal town/city that surrounds the Sarangani Bay Protected Seascape, Philippines.

The coastal areas of SarGen have gone through rapid changes over the years (de Jesus et al. 2001, Cabigas et al. 2012). There is approximately 514 ha of mangroves in SBPS following our estimate, in which the most extensive mangrove areas are on the east coast (Table 2). More than 60% of these are found in Glan, Malapatan, Alabel and General Santos City, while nearly 40% are on the west coast. Fig. 6 compares mangrove forests in different areas within SBPS using previous independent reports. The mangrove cover in SBPS was estimated in 1998 at 479 ha as such Maasim was lined by 152 ha of mangrove forests, the highest amongst all municipalities at that time (de Jesus et al. 2001). While in 2016, the mangrove forest cover of SBPS dropped to 332 ha (USAID Oceans 2019) and, in Maasim, it heavily shrank to only 29.73 ha 18 years later. Our estimate is also higher than the data presented by the DENR-FMB with 171 ha in the year 2010 (FMB 2012) and 328 ha in the year 2020 (FMB 2021). There are no mangrove cover data for General Santos City in these FMB reports. We used the data for South Cotabato since the city was part of the congressional representation of South Cotabato Province until 14 September 2021 and was the only coastal city of the Province.

No mangrove cover data were reported in Maitum in 1998 (de Jesus et al. 2001). In our measurement, Maitum has the largest area of mangrove forests within SBPS with 138 ha, a significant increase from only 28 ha recorded in 2016 (USAID Oceans 2019). Glan’s mangrove cover increased to 129 ha from 103 ha six years earlier. However, the extent of mangroves in Maasim, Kiamba and General Santos City did not change substantially from 2016 to 2022. Furthermore, these three areas have a low proportion of mangrove extent lengths in relation to the length of their coasts. Currently, the total extent of mangroves of SBPS has been estimated 35.46% higher than six years ago (Fig. 6, Suppl. material 4). This increase may be attributed to massive mangrove reforestation by the government, various civil society groups and other stakeholders (Gubalani 2021, Jumangit 2022) and community-based programmes that support sound coastal resource management (Calva 2018).

### Anthropogenic activities and threats

The growth and density of the human population adversely affect mangrove forests. The more people living in or near mangroves, the more anthropogenic impacts on the forests there will be (Alongi 2002). Rapid loss and degradation of forest cover have been reported in many mangrove ecosystems in large cities around the world (Branoff 2017). On the contrary, the fragmented mangrove forests in urban areas of Penang, Malaysia had more species and trees than the mangrove forests in rural areas. Around 40% of the total mangrove cover in 1990 was lost by 2000 in the Greater Bay Area of Guangdong, Hong Kong and Macao, mainly attributed to the increase in aquaculture ponds and built-up areas. However, it was observed that the mangrove area at the same site almost tripled after 18 years of conservation and restoration (Wang et al. 2021). Thus, mangrove forest structure is strongly determined by human actions and people can become partners in forest management (Walters 2004). Total tree cover is a rudimentary measure of environmental integrity. All else being equal, it may also indicate the capability and willingness of a political area to protect its natural environment, for example, in Tanalgo et al. (2022). A positive correlation between total tree cover and mangrove cover implies that, while forest trees are protected, mangrove deforestation is also prevented. The highest total tree cover and mangrove cover were in Maitum and Glan; therefore, they probably have the strictest regulations when it comes to protecting their biodiversity, while General Santos City and Maasim were low in both. General Santos City is leading in terms of economic growth in SarGen and, thus, the most able amongst areas to protect its natural environment. However, its mangrove forest cover remains low (Fig. 6, Suppl. material 4) while urban expansion continues. Fortunately, the city has been acting recently to protect and stabilise its shores (CMGC 2019, DENR 2021). While the mangrove and total tree covers of Alabel and Malapatan were relatively lower than in other municipalities, their proportions of mangrove forest extents relative to their coastal lengths were highest, indicating active and successful mangrove forest protection programmes implemented within their respective coastal territories.

**Figure 7.**
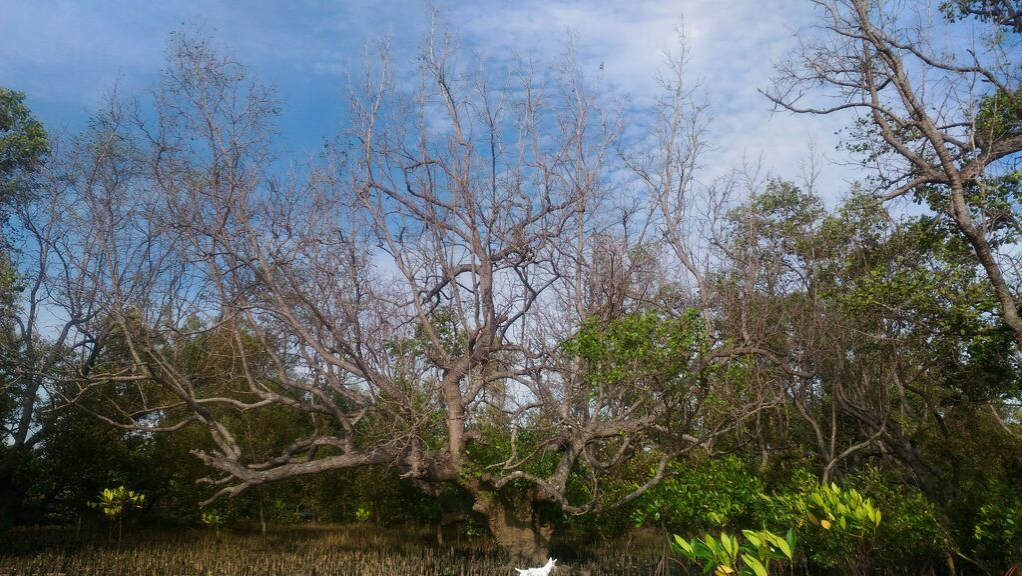
Defoliated *Sonneratiaalba* trees (Photo © MENRO, Alabel, Sarangani Province).

We found that the number of boats in SBPS was positively correlated with the total mangrove area (Fig. 4, Suppl. material 3). Camacho and Bagarinao (1986) also showed that mangrove cover was directly related to the number of fish landings, highlighting the support value of mangroves for local fisheries (Rönnbäck 1999). With increased mangrove cover, more economically important fishes and invertebrates thrive in the area and more local people are encouraged to venture into fishing. However, with a growing number of fishermen, fish catch also decreases (Santos et al. 2017). To compensate, the human population looks for alternatives to meet its consumption needs. Agriculture and aquaculture seem to be amongst the plausible solutions to reduce the gap between food supply and demand (Hashim et al. 2021), putting more pressure on mangrove ecosystems. Indeed, changes in land-use and -cover are amongst the strong forces driving mangrove forest loss in the world (Bunting et al. 2022), but differ in magnitude from country to country (Goldberg et al. 2020). It can lead to the failure to deliver ecosystem services and turn them from carbon (C) sinks to carbon sources contributing to global climate change (Donato et al. 2011, Alongi 2020, Harishma et al. 2020, Sasmito et al. 2020). Being at the interface of land and sea (Kumari et al. 2020) with large amounts of organic matter in their soils (Hossain and Nuruddin 2016), mangrove forests are a perfect place for agriculture and aquaculture production (Garcia et al. 2014). In Myanmar, rice cultivation has been an important driver of the decline in mangrove areas, while in Indonesia and Malaysia, the expansion of oil palm plantations resulted in the decrease of mangrove forest areas, whereas all of these activities have largely been held responsible for mangrove forest clearing in the Philippines (Richards and Friess 2016). In SBPS, onshore crops cultivated are mainly rice, corn and coconut. However, aquaculture farms, particularly for shrimp, are more widespread in the area. The worldwide loss of mangrove to aquaculture conversion between the 1970s, when the aquaculture industry started to flourish (Hashim et al. 2021) and 2009 was estimated at 544,000 ha or 28% of the total areal mangrove loss (Hamilton 2013), while 90% of the reported mangrove forest losses in the south and southeast Asia were caused by agriculture and shrimp farm developments (DasGupta and Shaw 2013). The aquaculture industry in SarGen is expanding even more. From 8000 metric tonnes in 2016, shrimp production in the area grew to 12,000 metric tonnes in 2018 from 850 ha operated by at least 35 growers and companies. Further expansion has been pushed to meet the increasing global demand (PNA 2018). This attempt poses additional potential threats to SBPS waters. Substances for soil and water treatment, such as lime and zeolite, growth inhibitors, such as antibiotics, disinfectants, pesticides and algicides and growth promoters including fertilisers, added vitamins and minerals in feeds are some of the chemicals used in shrimp farms in the Philippines (Primavera et al. 1993, Primavera 2006). Notwithstanding the unwanted effects of growth inhibitors on biodiversity and the environment (Chen et al. 2018, Olsvik et al. 2019, Pepi and Focardi 2021), fertilisers and other growth enhancers from aquaculture and agriculture sources cause eutrophication which leads to unwarranted algal growth, depleting oxygen, reducing water quality and endangering aquatic life (Streicher et al. 2021, Jwaideh et al. 2022). Moreover, wood smoke emission from charcoal production is one of the potential threats to mangroves observed in SBPS. Smokes have a high concentration of ethylene (Morgott 2015) which may cause physiological impairments, such as reduction of photosynthesis (Calder et al. 2010), induction of senescence and necrosis leading to plant death (Iqbal et al. 2017). Small-time charcoal factories were observed inside and nearby mangrove forests in some localities, which not only released smoke, but also had mangrove deforestation implications. A die-off of 40 trees of *S.alba* (Fig. 7) and one *A.marina*, making up an area of approximately 4,802 m^2^ in Kawas, Alabel, Sarangani Province took place in July 2018. We observed that only a specific portion of the forest was affected and it occurred on the upper part of the trees first and then progressed down. The possibility that the water quality, substrate characteristic, climatic condition, pesticide, insect infestation or disease as the cause of the die-off were excluded. However, about 25 m away from the back of the mangrove forest, a coconut shell charcoal factory was operating during night-time only, according to the residents. Thus, this defoliation event could be attributed to excessive smoke exposure coming from the nearby charcoal processing plant. Other observable evidence was the dying-off of the bananas around the factory, as well as the observable soot particles that were sticking to the bark of the mangroves.

### Mangrove-friendly approaches

To minimise mangrove loss problems, the adoption of an integrated mangrove-aquaculture production system known as silvoaquaculture or silvofisheries seems promising. It is a mangrove-friendly alternative to aquaculture pond development that can sustain not only productivity and livelihood, but also the conservation of mangrove ecosystems (Primavera et al. 2000, Susilo et al. 2018). It is a low-input farming system, which is mainly based on the harmonious interactions of marine and terrestrial resources (Udoh 2016) that form the biophysical condition of the mangrove forest. It was initially developed in Myanmar and later introduced in Indonesia in 1978 (Fitzgerald 2000, Takashima 2000). Although it has a few restrictions, other countries have embraced it and later introduced various models, including Nigeria (Akinrotimi et al. 2011, Udoh 2016), Malaysia, Philippines and Thailand (Primavera et al. 2000, Tanan and Tansutapanich 2000). In addition, utilising unproductive and abandoned aquaculture ponds for mangrove reforestation is another viable option (Wang et al. 2021), since they mostly lie in areas where mangroves had grown in the past (Stevenson et al. 1999). This strategy already worked in privately-owned abandoned fishponds of the Mallare clan in Nalus, Kiamba, Sarangani Province. The owners let the fishponds turn into a mangrove forest, now known as Mallare Mangroves. Today, mangroves thrive well in the area and the forest cover continues to expand, filling empty ponds with native mangroves. It is currently being established as a mangrove eco-park to help raise awareness of the socio-ecological importance played by mangroves and provide additional income for the local communities surrounding the mangrove site. The same has also been implemented in Leganes, Ilo-ilo, Philippines, now known as Katunggan Park, for the mitigation of climate change and has later become a tourist and learning destination as the result of the community-based mangrove rehabilitation programme of the local government unit of Leganes and Zoological Society of London-Philippines (Mayuga 2017).

This work generated the first comprehensive and current list of mangrove species diversity and a mangrove extent map for SBPS in the southern Philippines. Due to the sparse stature of the mangroves and patchy and fringing nature of the mangrove forests in SBPS, they are difficult to map using previously developed remote sensing models (Baloloy et al. 2020, Neri et al. 2021). Consequently, mangroves of SBPS are not receiving appropriate conservation attention compared to other mangrove forests in the country. Yet, a simple and practical method allowed us to provide valuable information about the mangrove areal extent in SBPS. Additionally, although we did not explore the degree of impacts of specific threats, we have provided a preview of potential threats to the mangroves of SBPS, particularly forest clearing, over-extraction and pollution. In-depth exploration addressing such a limitation is warranted for future research. Furthermore, we highlighted the value of expanding mangrove cover, the potential of mangrove-friendly aquaculture and the reforestation of degraded lands. To implement these successfully, we underscored the importance of understanding the causes of mangrove loss and the roles humans play in the dynamics of mangrove forest structure. These substantial results filled the knowledge gap about mangroves to guide future policies on the conservation and management of mangrove ecosystems within SBPS.

## Data resources

The georeferenced mangrove distributions can be accessed through the Global Biodiversity Information Facility (GBIF), https://doi.org/10.15468/pz5yp6 (Agduma and Cao 2023).

## Supplementary Material

DB3666D1-36F3-536D-9676-48DC25B623F0 10.3897/BDJ.11.e100050.suppl1 Supplementary material 1 Georeferenced locations of mangroves in Sarangani Bay Protected Seascape, Philippines Data type Occurrences of mangroves Brief description This data file contains the georeferenced locations (latitude, longitude) of mangroves in Sarangani Bay Protected Seascape (SBPS), Philippines, their IUCN and DENR conservation status and their occurrences in different towns surrounding SBPS. File: oo_827027.csv https://binary.pensoft.net/file/827027 Angelo Rellama Agduma, Kun-Fang Cao

16FA8340-2CC8-5406-AC9D-130B7277ADDE 10.3897/BDJ.11.e100050.suppl2 Supplementary material 2 Land use cover of coastal towns of Sarangani Province and General Santos City, Philippines Data type Measured land-use cover Brief description This data file summarises the measured land use cover (km^2^) of the towns surrounding Sarangani Bay Protected Seascape, Philippines, based on Sentinel-2 satellite data. File: oo_790406.csv https://binary.pensoft.net/file/790406 Angelo Rellama Agduma, Kun-Fang Cao

EC2BD7BB-6EB0-5655-860C-0B8F0FC6E5D2 10.3897/BDJ.11.e100050.suppl3 Supplementary material 3 Relationship (Spearman) of mangrove cover with total tree cover and proxies of potential threats to mangroves in Sarangani Bay Protected Seascape, Philippines Data type Correlation matrix (Spearman) Brief description This data file contains the areas of mangrove cover (ha) and of land-use cover (km^2^) (total tree cover, rangeland, cropland, built area, bare ground), the total population and the number of fishing boats in the coastal towns surrounding Sarangani Bay Protected Seascape, Philippines (Table 1). The results of correlation of mangrove cover with land-use cover, total population and number of fishing boats are emphasised in Table 2. File: oo_790408.docx https://binary.pensoft.net/file/790408 Angelo Rellama Agduma, Kun-Fang Cao

0C5EBD8F-1A78-5F12-B43E-8C3E41E61AF5 10.3897/BDJ.11.e100050.suppl4 Supplementary material 4 Mangrove cover of Sarangani Bay Protected Seascape, Philippines Data type Mangrove area Brief description This data file summarises the mangrove cover records (hectares) in the different coastal towns surrounding Sarangani Bay Protected Seascape, Philippines in 1998 (de Jesus et al. 2001), 2016 (USAID Oceans 2019) and 2022 (this study). File: oo_790087.csv https://binary.pensoft.net/file/790087 Angelo Rellama Agduma, Kun-Fang Cao

66C964B8-C514-5DDB-A94C-62D593D7BCE8 10.3897/BDJ.11.e100050.suppl5 Supplementary material 5 Confusion matrix for the generated extent map for mangroves of Sarangani Bay Protected Seascape, Philippines Data type Confusion matrix Brief description This is a confusion matrix containing the overall accuracy and Kappa coefficient that tell the validity of the mapping of mangrove areal extent used in the analysis. File: oo_812327.xls https://binary.pensoft.net/file/812327 Angelo Rellama Agduma, Kun-Fang Cao

## Figures and Tables

**Table 1.** List of true mangrove species documented in various sites within Sarangani Bay Protected Seascape, Philippines. The numbers indicate the reference sources: **1**: Barcelete et al. (2016), **2**: Bigsang et al. (2016), **3**: Jumawan (2022), **4**: Lagnason et al. (2016), **5**: Mullet et al. (2014), **6**: Natividad et al. (2014), **7**: Natividad et al. (2015), **8**: This study. Legend: **IUCN**: Red List of Threatened Species of the International Union for Conservation of Nature (IUCN 2022-1); **DENR**: Department of Environment and Natural Resources Updated National List of Threatened Philippine Plants and Their Categories (DAO 2017-11); **EN**: endangered, **VU**: vulnerable, **NT**: near-threatened, **LC**: least concern, **OWS**: other wildlife species; Site Codes: **ALA**: Alabel, **GLA**: Glan, **KIA**: Kiamba, **MAA**: Maasim, **MAI**: Maitum, **MAL**: Malapatan, **GES**: General Santos City.

Family	Species	IUCN	DENR	ALA	GLA	KIA	MAA	MAI	MAL	GES
Acanthaceae	*Acanthusebracteatus* Vahl	LC	OWS			8			5	
Acanthaceae	*Avicennialanata* Ridl.	VU	OWS	4						
Acanthaceae	*Avicenniamarina* (Forssk.) Vierh.	LC	OWS	3, 4, 6, 7, 8	1, 8		2, 6, 7, 8	8	1, 5, 8	8
Acanthaceae	*Avicenniarumphiana* Hallier f	VU	OWS		1, 8	8		8	1, 5, 8	
Arecaceae	*Nypafruticans* (Thunb.) Wurmb.	LC	OWS	8	8	8		8	5, 8	
Bombacaceae	*Camptostemonphilippinense* (S.Vidal) Becc.	EN	EN						8	
Combretaceae	*Lumnitzeralittorea* (Jack) Voigt.	LC	OWS				8		8	
Combretaceae	*Lumnitzeraracemosa* Willd.	LC	OWS	3, 6, 7, 8	8		8		5, 8	
Euphorbiaceae	*Excoecariaagallocha* L.	LC	OWS	8	8		8		5, 8	
Lythraceae	*Pemphisacidula* J.R. Forst. & G. Forst.	LC	EN	3, 6, 7, 8	8		6, 7		5, 8	
Meliaceae	*Xylocarpusgranatum* J.Koenig	LC	OWS	3, 6, 7		8	8		5, 6, 7	
Meliaceae	*Xylocarpusmoluccensis* (Lam.) M. Roem.	LC	OWS	3, 8		8	8		5	
Myrsinaceae	*Aegicerascorniculatum* (L.) Blanco	LC	OWS			8				
Myrsinaceae	*Aegicerasfloridum* Roem. & Schult.	NT	OWS	3, 4, 6, 7, 8					5, 8	
Rhizophoraceae	*Bruguieracylindrica* (L.) Blume	LC	OWS	3, 6, 7, 8	8		6, 7, 8		5	
Rhizophoraceae	*Bruguieragymnorrhiza* (L.) Lam.	LC	OWS	3, 6, 7, 8	1	8	2, 6, 7, 8		5	
Rhizophoraceae	*Bruguierasexangula* (Lour.) Poir.	LC	OWS		1			8		
Rhizophoraceae	*Ceriopszippeliana* (Griff.) Ding Hou	LC	OWS	3, 6, 7, 8			6, 7		5	
Rhizophoraceae	*Ceriopstagal* (Perr.) C.B.Rob.	LC	OWS	3, 4, 6, 7, 8	1, 8		8		1, 8	
Rhizophoraceae	*Rhizophoraapiculata* Blume	LC	OWS	3, 4, 6, 7, 8	1, 8	8	2, 6, 7, 8	8	1, 5, 8	8
Rhizophoraceae	*Rhizophoramucronata* Lam.	LC	OWS	3, 6, 7			2, 6, 7		1, 5	
Rhizophoraceae	*Rhizophorastylosa* Griff.	LC	OWS	8	8	8	8	8	8	8
Sonneratiaceae	*Sonneratiaalba* J. Smith	LC	OWS	3, 4, 6, 7, 8	1, 8	8	2, 8	8	1, 5, 6, 7, 8	8
Sonneratiaceae	Sonneratiacaseolaris (L.) Engl.	LC	OWS	8				8		
Total Species:	24	Species per town:		18	14	10	15	8	20	4

**Table 2.** Measured coastal length, mangrove extent and extent length of different coastal towns surrounding the Sarangani Bay Protected Seascape, Philippines.

Town/City	Coastal Length (km)	Mangrove Extent Length (km)	Mangrove Extent Length Proportion (%)	Mangrove Extent (ha)	Coastal length corrected mangrove area (ha/km)	Contribution (%)
Alabel	**10.24**	6.93	**67.66**	78.11	**7.63**	15.20
General Santos	28.30	3.95	13.95	36.85	1.30	7.17
Glan	59.60	11.07	18.57	128.76	2.16	25.05
Kiamba	39.96	2.56	6.40	37.24	0.93	7.24
Maasim	41.39	3.12	7.55	29.40	0.71	5.72
Maitum	21.11	**12.67**	60.01	**138.21**	6.55	**26.89**
Malapatan	17.58	9.11	51.80	65.46	3.72	12.74
SBPS (Total)	218.18	49.40	22.64	514.03	2.36	100.00
Note: Bold numbers emphasise the highest record for each item amongst coastal towns.						
