# Supplementary material for: Species richness, extent and potential threats to mangroves of Sarangani Bay Protected Seascape, Philippines
Source: Biodivers Data J. 2023 Mar 28;11:e100050. doi: 10.3897/BDJ.11.e100050 (PMC10848689; doi:10.3897/BDJ.11.e100050)
Supplement: Supplementary material 3 — Relationship (Spearman) of mangrove cover with total tree cover and proxies of potential threats to mangroves in Sarangani Bay Protected Seascape, Philippines [file bdj-11-e100050-s003.docx]

**Supplementary Table 1.** Summary of mangrove cover, land-use cover, population, and number of fishing boats in towns surrounding SBPS.

| **Towns** | **Mangrove cover (ha)** | **Total tree cover (km^2^)** | **Rangelands (km^2^)** | **Croplands (km^2^)** | **Built area (km^2^)** | **Bareground (km^2^)** | **Total population** | **Number of fishing boats** |
| --- | --- | --- | --- | --- | --- | --- | --- | --- |
| **Alabel** | 78.11 | 231.90 | 180.49 | 56.36 | 15.60 | 0.54 | 88294 | 167 |
| **General Santos City** | 36.85 | 150.90 | 113.01 | 126.35 | 127.13 | 1.01 | 697315 | 456 |
| **Glan** | 128.76 | 581.12 | 103.69 | 8.32 | 10.01 | 0.05 | 109547 | 1645 |
| **Kiamba** | 37.24 | 393.48 | 6.01 | 18.09 | 7.98 | 0.02 | 65774 | 835 |
| **Maasim** | 29.40 | 237.04 | 180.36 | 13.26 | 12.24 | 0.43 | 64940 | 655 |
| **Maitum** | 138.21 | 247.44 | 10.38 | 19.33 | 6.89 | 0.12 | 44185 | 1208 |
| **Malapatan** | 65.46 | 393.09 | 154.09 | 10.42 | 6.60 | 0.05 | 80741 | 663 |

**Supplementary Table 2.** Result of Spearman correlations between total mangrove cover, total tree cover and indicators of potential threats

| Environmental variables |  | Mangrove cover | Total tree cover | Rangelands | Croplands | Built area | Bareground | Total population | Number of fishing boats |
| --- | --- | --- | --- | --- | --- | --- | --- | --- | --- |
| Mangrove cover | Spearman's rho | — |  |  |  |  |  |  |  |
|  | p-value | — |  |  |  |  |  |  |  |
| Total tree cover | Spearman's rho | **0.393** | — |  |  |  |  |  |  |
|  | p-value | 0.396 | — |  |  |  |  |  |  |
| Rangelands | Spearman's rho | **-0.286** | -0.571 | — |  |  |  |  |  |
|  | p-value | 0.556 | 0.200 | — |  |  |  |  |  |
| Croplands | Spearman's rho | **-0.143** | -0.821 | 0.107 | — |  |  |  |  |
|  | p-value | 0.783 | 0.034 | 0.840 | — |  |  |  |  |
| Built area | Spearman's rho | **-0.429** | -0.679 | 0.429 | 0.571 | — |  |  |  |
|  | p-value | 0.354 | 0.110 | 0.354 | 0.200 | — |  |  |  |
| Bareground | Spearman's rho | **-0.286** | -0.964 | 0.643 | 0.714 | 0.714 | — |  |  |
|  | p-value | 0.556 | 0.003 | 0.139 | 0.088 | 0.088 | — |  |  |
| Total population | Spearman's rho | **-0.107** | -0.179 | 0.214 | 0.143 | 0.571 | 0.286 | — |  |
|  | p-value | 0.840 | 0.713 | 0.662 | 0.783 | 0.200 | 0.556 | — |  |
| Number of fishing boats | Spearman's rho | **0.536** | 0.857 | -0.750 | -0.643 | -0.643 | -0.786 | -0.286 | — |
|  | p-value | 0.236 | 0.024 | 0.066 | 0.139 | 0.139 | 0.048 | 0.556 | — |
